# EUS-FNA WITH 19 OR 22 GAUGES NEEDLES FOR GASTRIC SUBEPITHELIAL LESIONS OF THE MUSCLE LAYER

**DOI:** 10.1590/0102-672020180001e1350

**Published:** 2018-06-21

**Authors:** César Vivian LOPES, Antônio Atalíbio HARTMANN, Everson Luiz de Almeida ARTIFON

**Affiliations:** 1 Department of Gastroenterology and Digestive Endoscopy; 2 Department of Pathology, Santa Casa Hospital, Porto Alegre, RS;; 3 Department of Surgery, University of São Paulo School of Medicine, São Paulo, SP, Brazil

**Keywords:** Diagnosis, Endoscopic ultrasonography, Fine needle aspiration, Gastrointestinal stromal tumor, Leiomyoma., Diagnóstico, Leiomioma, Punção aspirativa com agulha fina, Tumor do estroma gastrointestinal, Ultrassonografia endoscópica.

## Abstract

**Background:**

Tissue diagnosis is required for gastric subepithelial lesions for differential diagnosis of GISTs. However, there has not been consensus about the best needle for EUS-guided sampling of these lesions.

**Aim:**

To evaluate the diagnostic yield of EUS-FNA for gastric subepithelial lesions of the proper muscle layer with large-bore 19 gauge needles.

**Methods:**

A prospectively maintained database was retrospectively reviewed to identify consecutive patients who underwent EUS-FNA with 19 and 22 gauge needles for gastric subepithelial lesions of the fourth endosonographic layer in a tertiary care referral center. EUS-FNA was performed by the same endosonographer, using the fanning technique, without on-site cytopathologist. Specimens were analysed through cell blocks by the same pathologist. Procedure results were categorized into diagnostic, defined as enough material for histopathology and immunohistochemistry, or nondiagnostic.

**Results:**

Eighty-nine patients (mean age: 59 years, 77% women) underwent 92 EUS-FNA with 19 (75) or 22 (17) gauge needles. Mean lesion size was 22.6 mm. Overall diagnostic yield was 88%. The diagnostic yield of 19 gauge was higher than that of 22 gauge needle (92%x70.6%; p=0.0410), and similar for lesions >2 cm and <2 cm (93.7%x90.7%; p=0.9563). The best performance for 19 gauge needles was obtained performing <3 needle passes. Complication rate was 2.8%.

**Conclusions:**

Diagnostic yield of EUS-FNA with 19 gauge needles is 92% for gastric subepithelial lesions of the proper muscle layer. It is safe and highly valuable for differentiation between GIST and leiomyoma, no matter the size of the lesion.

## RESUMO

**Racional:**

O diagnóstico tecidual é mandatório nas lesões subepiteliais gástricas da camada muscular própria para o diagnóstico diferencial das neoplasias do estroma gastrointestinal (GISTs). Contudo, ainda não há consenso quanto a melhor agulha para a punção ecoguiada destas lesões.

**Objetivo:**

Avaliar o valor da punção aspirativa ecoguiada com agulhas calibrosas de 19 gauge para o diagnóstico diferencial das lesões subepiteliais gástricas da camada muscular própria.

**Métodos:**

Foram revisados retrospectivamente os registros de pacientes consecutivos submetidos à punção aspirativa ecoguiada com agulhas de 19 e 22 gauge de lesões subepiteliais gástricas da quarta camada ecográfica em um centro de referência. A punção aspirativa foi realizada sempre pelo mesmo endoscopista, com o emprego da técnica de fanning, sem presença de citopatologista em sala. O material aspirado foi avaliado apenas pela técnica de cell block pelo mesmo patologista. Os resultados foram considerados diagnósticos, na presença de material adequado para coloração pela H&E e imunoistoquímica, ou não-diagnósticos.

**Resultados:**

Oitenta e nove pacientes (idade média: 59 anos, 77% do sexo feminino) foram submetidos a 92 punções aspirativas ecoguiadas com agulhas de 19 (75) ou 22 (17) gauges. O tamanho médio das lesões foi de 22,6 mm. O ganho diagnóstico geral foi de 88%. O ganho diagnóstico para as agulhas de 19 gauge foi superior ao das agulhas de 22 gauge (92%x70,6%; p=0,0410), e similar para lesões >2 cm e <2 cm (93,7%x90,7%; p=0,9563). Os melhores resultados com a agulha de 19 gauge foram obtidos com a realização de até três punções. A taxa de complicações foi de 2,8%.

**Conclusão:**

O ganho diagnóstico da punção aspirativa ecoguiada de lesões subepiteliais gástricas da camada muscular própria com agulhas de 19 gauge é de 92%. A punção com a agulha mais calibrosa para lesões de qualquer tamanho é procedimento seguro e de grande valor no diagnóstico diferencial dos GISTs e leiomiomas.

## INTRODUCTION

Endoscopic ultrasonography-guided fine-needle aspiration (EUS-FNA) is a minimally invasive technique for sampling gastric subepithelial lesions (SELs), which are a challenge for the differential diagnosis of gastrointestinal stromal tumors (GISTs). The yield of EUS-FNA for diagnosis of these lesions ranges from 49-73% with 22 gauge needles^1^ ^,^ ^9^ ^,^ ^16^, but often the specimens are insufficient for immunostaining. Regarding the needle size, the literature about large-bore needles is too limited. 

The objective of this study was to review the results of EUS-FNA with 19 gauge needles for gastric SELs of the proper muscle layer performed under the same routine technique, and with specimens evaluated through cell blocks. 

## METHODS

### Study design

**FIGURE 1:**
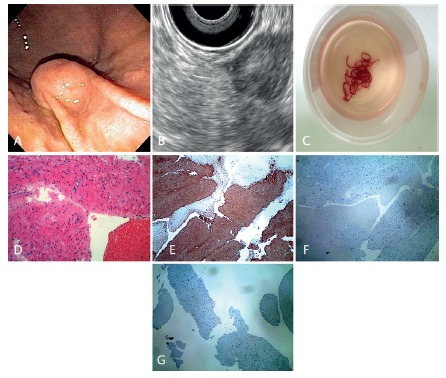
A) Gastric subepithelial lesion from the greater curvature of the body; B) linear EUS array demonstrating a lesion from the proper muscle layer; C) EUS-FNA specimens after a total of three needle passes with a 19 gauge needle; D) histopathology confirming a spindle cell tumor (H&E); E) immunohistochemistry stain positive for actin; F) negative for c-kit; G) DOG-1, confirming a gastric leyomioma.

Eligible patients included those referred for EUS-FNA at a single tertiary-referral center. Inclusion criteria were patients with hypoechogenic gastric SELs of the proper muscle layer (Figures 1A e 1B). Exclusion criteria were an INR>1.5 or platelet count <50,000, lesions from the submucosa (ectopic pancreas) and cysts. The first 25 EUS-FNA of SELs were also excluded, of which 18 gastric and seven esophageal SELs, in order to reach the minimum number of EUS-FNA procedures before competency can be assessed according to the guidelines from American and European Societies of Gastrointestinal Endoscopy^7^ ^,^ ^20^. All patients signed informed consent before enrollment.

### EUS-FNA technique

All procedures were performed by the same endosonographer with a curvilinear array echoendoscope (Olympus GF-UCT140-AL5, Olympus America Inc., New York, USA), coupled to an ultrasound unit Aloka Prosound alfa-5 SX. Needles for EUS-FNA were 19 or 22 gauge (EchoTip Ultra Echo-19 or 22, Cook Medical, Winston-Salem,USA) until July 2015, and only 19 gauge needles after that time. EUS-FNA was performed under deep sedation with the assistance of an anesthesiologist. The needle was advanced under EUS guidance into the target lesion, the stylet was removed, 10 ml of suction was applied, and the needle was moved back and forth 10 to 20 times in a fan-like motion within the lesion during each needle pass. After removal of the needle, the specimens were placed in 20% buffered formalin. The specimens were regarded as adequate in the presence of whitish cores (tumor tissue) and reddish cores (coagula with tumor tissue, Figure 1C). On-site cytopathologist was not available. No smears were prepared. Patients were monitored for 1 h after the procedure.

### Pathology 

The histological analysis of the specimens were made through cell blocks by the same experienced gastrointestinal pathologist. The material was stained with H&E (Figure 1D), and immunohistochemistry stain for actin antibodies, c-kit, and DOG-1 was performed in the presence of spindle cells lesions (Figures 1E, 1F and 1G). A specimen was defined as diagnostic when sufficient for histopathologic evaluation and immunohistochemistry analysis. If the biopsies were insufficient for complete evaluation, the specimens were considered non-diagnostic. 

### Statistical analysis

Categorical variables were compared by chi-square test or Fisher’s exact test. Continuous variables were compared by Student’s t-test. Statistical analyses were performed using SPSS software (version 15.0, SPSS, Chicago, IL).

## RESULTS

### Patients demographics

From September 2009 to January 2017, a total of 129 patients who underwent 132 EUS-FNA procedures were studied. Twenty-two lesions were excluded from the analysis, of which 13 submucosal lesions and nine duplication cysts. After excluding the first 18 EUS-FNA of gastric SELs, the final study group was composed of 89 patients with hypoechogenic gastric SELs of the proper muscle layer, which were submitted to 92 EUS-FNA with 19 (n=75) or 22 gauge (n=17) needles. Repeated EUS-FNA procedures were performed in two patients. The baseline characteristics of the patients and lesions are summarized in Table 1. 

**TABLE 1:** Demographics and characteristics of the patients/lesions

	TOTAL	19 gauge needles	22 gauge needles	p
n	89	72	17	
Sex (F/M)	69 / 20	57 / 15	12 / 5	
Age, mean + SD (range), yr	58.7 + 14.5 (17-94)	59.2 + 13.5 (25-94)	56.3 + 18.4 (17-86)	0.4611
EUS-FNA	92	75	17	
Size, mean + SD (range), mm	22.6 + 18.6 (5-140)	23.8 + 19.7 (6.5-140)	17.4 + 12.0 (5-50)	0.2027
Size > 2 cm	34	32	2	
Size < 2 cm	58	43	15	
Needle passes, mean + SD (range)	2.9 + 1.13 (1-6)	2.8 + 1.0 (1-5)	3.4 + 1.41 (1-6)	0.0341
< 3 needle passes	69	59	10	
> 3 needle passes	23	16	7	
Diagnosis	81 (88%)	69 (92%)	12 (70.6%)	0.0410
GIST	38	36	2	
Leiomyoma	41	32	9	
Schwannoma	1	1	0	
Adenocarcinoma/linitis	1	0	1	
Complications	2 (2.2%)	2 (2.8%)	0	

### Diagnostic yield of EUS-FNA: 19 x 22 gauge needles

Needle punctures were successful in all cases irrespective of lesion location. The overall diagnostic yield of EUS-FNA was 88% (81/92). The diagnostic yield of EUS-FNA with 19 gauge needles was higher than that of 22 gauge needles [92% (69/75)x70.6% (12/17); p=0.0410]. 

### Diagnostic yield of EUS-FNA with 19 gauge needles according to the lesion size and number of needle passes 

EUS-FNA with 19 gauge needles revealed the same diagnostic yield for lesions >2 cm and <2 cm [93.7% (30/32)x90.7% (39/43); p=0.9563].

The mean number of needle passes for gastric SELs of the proper muscle layer with 19 gauge needles was 2.8+1. For lesions >2 cm and <2 cm, the mean number of needle passes were, respectively, 2.84+0.95 and 2.72+1.09, with median of three needle passes. The diagnostic yields for EUS-FNA with 19 gauge needles were, respectively, 98.3% (58/59) and 68.7% (11/16) when performing <3 or >3 needle passes (p=0.00082). 

The diagnostic yields of EUS-FNA for lesions >2 cm with 19 gauge needles were similar when performing <3 or >3 needle passes [96.1% (25/26)x83.3% (5/6); p=0.8145]. On the other hand, for lesions <2 cm, EUS-FNA with 19 gauge needles and <3 needle passes offered the best diagnostic yield [100% (33/33)x60% (6/10); p=0.0014]. 

### Complications 

The complication rate was very low. Two (2.8%) cases developed epigastric pain due to hematoma of the gastric wall after EUS-FNA with 19 gauge needles, one of them with a 5 cm exofitic GIST requiring surgical intervention. The other case was managed conservatively, with no need of blood transfusion.

## DISCUSSION

In our experience, EUS-FNA was performed with 19 gauge needles for 75 gastric SELs of the proper muscle layer, which represents the largest study published to date with this needle for this kind of lesions. Its diagnostic yield was higher than that of 22 gauge needles (92%x70.6%; p=0.0410), and revealed results higher than 90% despite the size of the lesions. Its diagnostic yields were, respectively, 98% and 68.7% when performing <3 or >3 needle passes (p=0.00082).

Concerning the different types of needles for EUS-guided sampling of SELs, Zhang *et al*.^24^ did not demonstrate difference in diagnostic rate for any kind of needle. However, among 17 studies included in this meta-analysis, 14 used EUS-FNA needles, but only three with 19 gauge needles^6^ ^,^ ^18^ ^,^ ^22^, corresponding to less than 9% of the evaluated cases. Other seven studies evaluated EUS-FNB needles, five with trucut needles^5^ ^,^ ^8^ ^,^ ^9^ ^,^ ^14^ ^,^ ^19^, and two with a core trap needle (ProCore^®)^)^10^ ^,^ ^12^. There was only five comparative studies^4^ ^,^ ^8^ ^,^ ^11^ ^,^ ^12^ ^,^ ^22^, and only one of them evaluating EUS-FNA with 19 gauge needles^22^. The number of evaluated cases for different types of needles was very small in six studies, each one with less than 20 cases for every type of needle^2^ ^,^ ^4^ ^,^ ^9^ ^-^ ^12^. In regard to the type of SELs, six of 14 studies were not restricted to gastric SELs^2^ ^,^ ^5^ ^,^ ^9^ ^,^ ^12^ ^,1822^, and three other studies were not restricted to SELs, but also included lesions from other organs, especially pancreatic ones^4^ ^,^ ^10^ ^,^ ^11^. In reference to the wall layer evaluated, 10 of 17 studies were not restricted to the proper muscle layer^1^ ^,^ ^2^ ^,^ ^6^ ^,^ ^8^ ^,^ ^12^ ^,^ ^14^ ^,^ ^16^ ^,^ ^18^ ^,^ ^19^ ^,^ ^22^, this information was unclear in four studies ^4^ ^,^ ^10^ ^,^ ^11^ ^,^ ^23^, and only three analyzed specifically the proper muscle layer^5^ ^,^ ^9^ ^,^ ^20^, but two of them were not restricted to gastric SELs^5^ ^,^ ^9^, and none of them evaluated EUS-FNA with 19 gauge needles. At last, relating to histopathology, cell blocks were used in only nine (53%) studies^1^ ^,^ ^2^ ^,^ ^4^ ^,^ ^8^ ^,^ ^9^ ^,^ ^10^ ^,^ ^16^ ^,^ ^21^ ^,^ ^22^, eight of them evaluating EUS-FNA ^1^ ^,^ ^2^ ^,^ ^4^ ^,^ ^8^ ^,^ ^9^ ^,^ ^16^ ^,^ ^21^ ^,^ ^22^, but only a single study with 19 gauge needles^22^. This way, with significant heterogeneity among the selected studies, the best needle for EUS-guided sampling of gastric SELs of the proper muscle layer has not been already defined.

The experience already published for the EUS-FNA with 19 gauge needles for gastric SELs is constituted of four studies. In the experience by Larghi *et al*.^13^, using a forward-viewing linear echoendoscope, adequate specimens for histological examination and immunohistochemistry were obtained in 93% of the cases. Our results were very similar to that study, but we used a curvilinear array echoendoscope. Watson *et al.* ^22^ provided adequate specimens for diagnosis in 79% of the cases. This group counted on on-site cytopathologist in 65% of the procedures, and cell block was used. The diagnostic yields for SELs >20 and <20 mm were, respectively, 80% and 45%, but this difference was not significant in multivariate analysis. Besides, EUS-FNA with 19 gauge needles and a higher number of needle passes were not associated with improved yield. In our experience, we demonstrated better results with the large-bore needle, and the best yield was obtained with up to three needle passes. Eckardt *et al*.^6^, without on-site cytopathologist, using a combined evaluation with cyto and histopathology, with median lesion size of 24 mm, and an average number of two needle passes, obtained diagnostic material in 52% of the cases. Nonetheless, this material allowed immunohistochemistry stain in 91% of the cases. Unlike our study, these authors evaluated gastric SELs of the proper muscle layer in only 61% of the cases, which could explain the high rate of non-diagnostic cases, in spite of the needle caliber, lesion size, and number of needle passes have been similar to ours. At last, a study evaluating the specimens by means of cytopathology, without on-site cytopathologist, obtained adequate material in only 58% of the cases^17^. The 19 gauge needle may obtain a hemorrhagic specimen, which can difficult or even make unfeasible the cytopathologic evaluation. This needle must be used if the intention is to obtain tissue cores, and not only a group of cells.

Our diagnostic yield with a large-bore needle, with no restriction regarding the lesion size, with three or less needle passes, without on-site cytopathologist, with specimens evaluated through cell blocks is higher than that obtained with trucut needles, and as good as that obtained with Procore^®)^ needles. Beshir *et al*.^3^ comparing the trucut needle (EUS-TCB) to the EUS-FNA, demonstrated a diagnostic yield of EUS-TCB and EUS-FNA for SELs of the proper muscle layer of 64.5% and 66%, respectively. The literature is scarce on comparative studies between EUS-TCB and EUS-FNA with 19 gauge needles. Nevertheless, it is unlikely a study like this to be undertaken, as it is well know the higher incidence of technical failures which not allow the puncture in up to 15% of the cases^8^ ^,^ ^14^ ^,^ ^17^ ^,^ ^19^, and the absence of higher diagnostic yield even when compared to EUS-FNA with smaller caliber needles^8^. Concernig the Procore® needles, Kim *et al*.^12^ conducted a study comparing EUS-FNB to EUS-FNA, both with 22 gauge needles, for SELs >2 cm, neither restricted to the stomach nor to the proper muscle layer. The Procore^®)^ needle established the diagnosis with fewer number of passes, with median of two passes, and revealed an important difference in the diagnostic yield (92%x30%). However, the literature has not any comparative study between EUS-FNB with Procore^®)^ needles and EUS-FNA with 19 gauge needles for gastric SELs of the proper muscle layer.

Our complication rate was very low (2.8%) for EUS-FNA with 19 gauge needles. This rate is a little higher than the bleeding of 2.2% described by Eckardt *et al*. ^6^, but lower than 8% described by Na *et al*.^17^ with 22 gauge needles. With a better diagnostic yield, our complication rate is lower than the rates of 3-4% for trucut needles^14^ ^,^ ^17^ ^,^ ^19^, and there has not been complication report with Procore^®)^ needles for SELs.

This study is subject to the limitations inherent to its retrospective design, and the experience of a single endosonographer in solely a referral center. Furthermore, the diagnoses obtained by means of EUS-FNA were not compared to surgery^15^. However, as most patients were asymptomatic and their median lesion size was 16 mm, it would not be possible to submit all GISTs to resection. 

On the other hand, this study has many strengths as well. After an initial experience with 22 gauge needles, all EUS-FNA procedures were performed with 19 gauge needles for all gastric SELs of the proper muscle layer despite their presumptive EUS diagnosis, location and size. EUS-FNA with 19 gauge needles obtained a definitive diagnosis in most cases. The routine histopathology processing for the specimens was the same, and a single experienced gastrointestinal pathologist evaluated the material. The small sample sizes of previous studies, inclusion of suspected diagnoses, SELs from various sites of the gastrointestinal tract, and only lesions >2 cm might have led to an overestimation of the diagnostic yield of different needles in those studies. With a scarcity of studies comparing the yield of EUS-FNA for gastric SELs of the proper muscle layer using different needle calibers, our experience with 19 gauge needles is the largest when compared to other studies. We found a significant higher diagnostic yield with the 19 gauge needle even in the absence of on-site cytopathologists.

 The question about the best needle for EUS-guided biopsy for these lesions is still unclear. Further comparative, randomized and multicentric studies are necessary to define whether this approach is the best and most cost-effective diagnostic strategy for gastric SELs of the proper muscle layer. 

## CONCLUSION

Endoscopic ultrasonography-guided fine-needle aspiration of subepithelial gastric lesions of the own muscular layer in the absence of cytopathologist in the room, with up to three punctures with 19 gauge needles and evaluation of the material through cell blocks, allows a diagnostic gain of more than 90%. It is safe and highly valuable for differentiation between GIST and leiomyoma, no matter the size of the lesion. 
